# Gum-Saline in Wound-Shock and Similar States

**Published:** 1919-05-10

**Authors:** W. M. Bayliss

**Affiliations:** University College, London.


### THE EDITOR'S LETTER-BOX.

### Gum-Saline in Wound-Shock and Similar States.

*To the Editor of* THE HOSPITAL.

SIR,—In the otherwise excellent account of the discussion on wound-shock at the recent meeting of the B.M.A., given in your issue of April 26, it appears to me that the third paragraph is apt to convey a wrong impression of the present state of opinion on the use of gum-saline. The question is rather as to whether this solution is capable of replacing blood, even if less effectively, under circumstances in which it is impossible to perform transfusion of blood. No surgeon or physiologist would hold that it is superior to blood, but many who had large experience at the front state that they found the two fluids practically equivalent. Others, however, obtained the impression that in certain conditions, difficult to define, blood was superior. So far as I can discover none who used properly prepared solutions deny the value of gum-saline.

I venture to call attention to these facts on account of the possibility of a valuable method in civil practice being overlooked. It would be appropriate when cases of deficiency of blood in circulation from any cause, whether hæmorrhage, shock or toxæmia, require treatment. Further experience is eminently desirable, but it has recently come to my notice that Dr. Burkitt, of Nairobi, has restored hopeless cases of black-water fever by intravenous gum-saline, and there is reason to believe that cholera will receive similar benefit. A new field seems to be opened up here in the maintenance of an effective circulation to enable the body to throw off the disorder. In cholera, for example, the blood volume is greatly reduced by loss of fluid from the intestine, and it may be that the serious state is greatly due to deficient blood supply to vital organs.—I am, etc.,

W. M. BAYLISS.

May 1, 1919. University College, London.

